# Unraveling the Enigma: Correlating Thrombus Histopathology With the Number of Passes in Mechanical Thrombectomy

**DOI:** 10.7759/cureus.77994

**Published:** 2025-01-26

**Authors:** Namitha K Baby, Joel Sabu, Kevin Jose Madapat, Zulkifli Misri

**Affiliations:** 1 Internal Medicine, Kasturba Medical College, Mangalore, IND; 2 Internal Medicine, Father Muller Medical College, Mangalore, IND; 3 Clinical Pharmacy, JSS College of Pharmacy, Mysuru, IND; 4 Neurology, Kasturba Medical College, Mangalore, IND

**Keywords:** clot, ischemic stroke, mechanical thrombectomy (mt), number of passes, thrombus histopathology

## Abstract

Background

Mechanical thrombectomy has revolutionized the treatment of acute ischemic stroke. Although a few studies have explored the correlation between thrombus histopathology and the number of passes required, the relationship remains unclear. The composition of the thrombus significantly influences the complexity of the procedure. Research has shown that erythrocyte-rich clots are associated with better reperfusion outcomes and fewer passes, whereas fibrin-rich clots are more challenging to retrieve and yield poorer outcomes. This study aims to investigate the association between thrombus histopathology and the number of passes during mechanical thrombectomy.

Methods

This retrospective observational study included 60 patients undergoing mechanical thrombectomy. Thrombus samples were analyzed histologically using hematoxylin-eosin staining and classified as either erythrocyte-rich (>50% erythrocytes) or fibrin-rich (>50% fibrin). The number of thrombectomy passes and patient demographics were recorded. Statistical analysis was performed to identify associations.

Results

RBC-rich thrombi were associated with fewer passes (p=0.035). Additionally, patients aged 45-65 years had a higher proportion of fibrin-rich clots, which required more passes (p=0.021).

Conclusion

This study demonstrates a significant association between thrombus histopathology and the number of passes during mechanical thrombectomy. Understanding thrombus composition may aid in tailoring therapeutic approaches and improving patient outcomes. Overall, thrombus composition was significantly correlated with the number of passes, with erythrocyte-rich thrombi requiring fewer attempts for successful retrieval.

## Introduction

As per the definition put forward by the World Health Organization (WHO), "stroke is rapidly developing clinical signs of focal (or global) disturbance of cerebral function, with symptoms lasting 24 hours or longer, or leading to death, with no apparent cause other than of vascular origin" [1]. With a yearly death rate of around 5.5 million, stroke is positioned as the second major cause of death globally [2]. Most strokes are ischemic (87%), occurring due to the blockage of cerebral blood vessels by thrombi [3]. Because of the revelation of positive thrombectomy trials in 2015, endovascular procedures have been developed to mechanically retrieve thrombi from cerebral vessels [4-8]. Mechanical thrombectomy aims to achieve reperfusion as early as possible to enhance the likelihood of better functional outcomes for patients [9]. The thrombi retrieved by mechanical thrombectomy are accessible for histopathological examination, thus opening the door to a new field within stroke pathology. Detailed examination of stained slides has remained the foundation of histopathological analysis and diagnostic medicine for over a century. Studying the histopathology of recovered thrombi is pivotal to enhancing our understanding of the variability in thrombus composition across different etiologies [10]. The procedure may require numerous attempts or passes to retrieve the obstructing clot, consequently increasing the likelihood of unsatisfactory patient outcomes. Previous studies have shown that first-pass complete reperfusion strongly correlates with higher rates of favorable results, lower mortality, and fewer procedural adverse events [11-14]. 

Studies focusing on the number of attempts during interventional therapy have highlighted that the number of attempts is significant in predicting effective results in individuals suffering from acute ischemic stroke [11,15-19]. Successful target-vessel recanalization was identified as the most significant determinant of a better prognosis [17,20]. The composition of the clot might also predict the reperfusion rate. Erythrocyte-rich clots were linked with more favorable reperfusion rates [21], a lower chance of fragmentation [22], and required fewer passes for thrombus retrieval [23,24]. Erythrocyte-dominant clots have higher viscosity and deformability and lower elasticity and stiffness [25,26]. These consolidated features may enhance the efficacy of thrombectomy in recovering this type of thrombus. With each additional thrombectomy device pass, the reperfusion rate for uninterrupted thrombectomy consecutively decreases. The average number of attempts for the procedure is three; attempts beyond three do not enhance the chance of reperfusion, are not prognostic, and raise the possibility of complications such as hemorrhagic transformation [27]. Hence, a better prognosis is associated with fewer passes needed to achieve complete reperfusion of target vessels. Defining the relationship between the histopathology of thrombi and the number of passes may, in the future, be necessary for instrument selection, improving the methodological approach, and aiding the development of technologies to recover tough clots effectively. Nearly 30% of procedures achieving complete recanalization and reperfusion did not result in better patient outcomes [28]. The rationale behind clinically ineffective interventions for acute ischemic stroke remains unclear, perhaps due to an incomplete understanding of the pathophysiology.

Various studies have examined the nature of clots recovered from mechanical thrombectomy. It appears that most studies to date utilized software and Martius Scarlet Blue stain [29], which is rarely used in clinical settings for pathological examination in the Indian environment. Studies based on clinically obtained histological reports are scant. This study aims to recognize the histopathological characteristics of thrombi retrieved during the first pass and multiple passes in a mechanical thrombectomy procedure, which are then stained using hematoxylin-eosin.

The primary aim of this study is to find the association between the nature of thrombi recovered and the number of passes during the mechanical thrombectomy procedure. The thrombi analyzed will be categorized as either erythrocyte-rich (more than 50%) or fibrin-rich (more than 50%). The number of passes for each patient is obtained from the reports. Establishing the association between the number of passes and histopathological components in the future is crucial for equipment selection, improving thrombectomy methods, developing technologies to retrieve difficult thrombi successfully, and enabling clinicians to foresee the nature of thrombi before treatment, thereby modifying the therapeutic process accordingly.

## Materials and methods

Study design and setting

This study was conducted as a retrospective observational analysis at a tertiary care hospital, focusing on patients treated for acute ischemic stroke caused by large-vessel occlusion. The study spanned two years, from January 2020 to January 2022. It aimed to investigate the histopathological composition of thrombi retrieved during mechanical thrombectomy and its association with procedural factors, particularly the number of retrieval passes required to achieve successful recanalization. Mechanical thrombectomy is a well-established treatment for large-vessel occlusions, offering significant improvement in clinical outcomes for eligible patients. However, the relationship between the histopathological nature of thrombi and procedural efficiency remains underexplored. This study was designed to provide insights into these associations, with the potential to guide procedural strategies and improve patient outcomes.

Study population

A total of 60 patients who underwent mechanical thrombectomy within the study period were included in the analysis. These patients represented cases where thrombus retrieval was successful, and adequate samples were available for histopathological examination.

Inclusion criteria

The inclusion criteria included patients with confirmed large-vessel occlusion who underwent mechanical thrombectomy, cases where thrombus retrieval was successful and adequate samples were preserved for histopathological analysis, and early thrombectomy (patients who underwent thrombectomy within six hours of symptom onset).

Exclusion criteria

The exclusion criteria included patients in whom thrombus retrieval was unsuccessful, cases where the retrieved thrombus samples were insufficient or improperly preserved for analysis, and late thrombectomy (patients who underwent thrombectomy beyond six hours of symptom onset).

By restricting the analysis to these cases, the study ensured the availability of complete clinical, procedural, and histopathological data, thereby enhancing the reliability of the findings.

Ethical considerations

The study was conducted in compliance with the ethical standards outlined by the Declaration of Helsinki. Approval was obtained from the Institutional Ethics Committee, Kasturba Medical College, Mangalore, India (IEC KMC MLR 04-2022/110), before the study began. Given the retrospective nature of the analysis, no direct patient interaction was involved, and data collection was based on existing medical records. To ensure confidentiality, all patient identifiers were anonymized before data analysis. Patient data were handled with strict adherence to ethical guidelines, safeguarding privacy and maintaining data security throughout the study.

Data collection

Data were systematically collected using a pretested and validated semi-structured proforma specifically designed for the study objectives. This proforma included sections for capturing demographic details, procedural information, and histopathological findings.

Demographic Data

Basic patient characteristics, including age, gender, and relevant clinical history, were recorded to comprehensively describe the study population.

Procedural Data

Data on the number of retrieval passes required during mechanical thrombectomy were collected from radiological and procedural records. The number of passes served as a key procedural variable in the study.

Histopathological Data

Information regarding the histopathological nature of the retrieved thrombi was obtained from pathology department reports.

Thrombus Retrieval and Processing

During the mechanical thrombectomy procedure, thrombi were retrieved using standard thrombectomy devices, such as the RED 72 aspiration catheter (Penumbra, Inc., Alameda, California) and Solitaire X stent retriever (Medtronic, Dublin, Ireland). Immediately after retrieval, the thrombi were preserved in 10% neutral buffered formalin to maintain their structural integrity. The preserved samples were then transported to the histopathology department for further processing.

Sample Processing

Two sets of thrombus fragments were prepared for each patient. The samples were embedded in paraffin, sectioned into thin slices, and stained using hematoxylin and eosin (H&E) to enhance the visualization of cellular and extracellular components.

Histopathological Analysis

The stained sections were examined manually under a light microscope by experienced pathologists who were blinded to clinical information. The thrombi were classified based on their predominant histological components into the following categories: (1) erythrocyte-rich thrombi (Red Thrombi), characterized by a high proportion of red blood cells, and (2) fibrin-rich thrombi (White Thrombi), predominantly composed of fibrin with fewer red blood cells.

Statistical Analysis

All collected data were entered into the IBM SPSS Statistics for Windows, Version 28 (Released 2021; IBM Corp., Armonk, New York) for analysis. The latest version of SPSS was used to ensure compatibility and accuracy in data handling. Continuous variables, such as patient age, were summarized using means and standard deviations. Categorical variables, including gender and thrombus composition, were expressed as frequencies and proportions. To examine the relationship between the histopathological composition of thrombi and the number of retrieval passes required during thrombectomy, the chi-square test (χ²) was applied. This statistical test was chosen due to its suitability for categorical data analysis. A p-value of less than 0.05 was considered statistically significant, indicating a meaningful association between thrombus composition and procedural efficiency.

## Results

Among the 60 patients who underwent mechanical thrombectomy and whose thrombus was successfully retrieved, 40 (66.7%) were males, and 20 (33.3%) were females (Table 1), with a mean age of 55 ± 12.72 years. RBC-rich thrombus was present in 26 (43.3%) patients, of whom 17 were males and nine were females. The remaining 34 (56.7%) patients, of whom 23 were males and 11 were females, had fibrin-rich thrombi (Table 2). The optimum number of passes in our study was three, with a mean value of 3.27 ± 1.35 (Table 3). Out of 60 patients, 20 (33.3%) required three passes. The highest number of passes was eight, which occurred in one out of the 60 patients.

**Table 1:** Demographic and thrombus characteristics of patients undergoing mechanical thrombectomy

Variable	Frequency	Percentage
Gender		
Male	40	66.7
Female	20	33.3
Nature of thrombus		
RBC-rich	26	43.3
Fibrin-rich	34	56.7
Number of passes		
≤2	19	31.7
>2	41	68.3

**Table 2:** Statistical analysis of associations between thrombus characteristics, age, gender, and number of passes

Variables	Nature of thrombus		Chi-square	P-value
	RBC-rich	Fibrin-rich		
Gender				
Male	17	23	0.034	0.854
Female	9	11	0.034	0.854
Age				
25-45 years	9	4	7.75	0.021
45-65 years	10	25	7.75	0.021
65-85 years	7	5	7.75	0.021
Number of passes				
≤2	12	7	4.45	0.035
>2	14	27	4.45	0.035

**Table 3:** Distribution of number of passes during mechanical thrombectomy

Variables	Mean±SD	Median	Mode
Age	55±12.72	57.5	59
Number of passes	3.27±1.35	3	3

Two patients achieved successful reperfusion with a single pass (Table 4). We categorized the number of passes into two groups: the first group included patients who achieved successful reperfusion with one or two passes, while the second group included those who required three to eight passes. Out of the 60 patients, 19 (31.7%) belonged to the first group (≤2 passes), while the remaining patients were in the second group (>2 passes) (Table 1). We sought to determine whether there was any association between the nature of the thrombus and variables such as gender, age, and the number of passes by calculating the chi-square statistic and the corresponding p-value. A significant association was considered present only if the p-value was less than 0.05 (Table 2). The chi-square value for the association between the nature of the thrombus and gender was 0.034, and the p-value was 0.854, indicating no significant association between the nature of the thrombus and gender.

**Table 4:** Distribution of patients by number of passes required for mechanical thrombectomy

Number of passes	Frequency	Percentage
1	2	3.3
2	17	28.3
3	20	33.3
4	11	18.3
5	6	10
6	3	5
8	1	1.7

The age was grouped into three categories: 25-45 years, 45-65 years, and 65-85 years. Within the age group of 25-45 years, nine patients had RBC-rich thrombi, and four had fibrin-rich thrombi. In the 45-65 years age group, 10 had RBC-rich thrombi, and 25 had fibrin-rich thrombi, making this group the largest. In the 65-85 years age group, seven were RBC-rich, and five were fibrin-rich. The chi-square value for the association between the nature of the thrombus and age was 7.75, with a p-value of 0.021, indicating a significant association between age and the nature of the thrombus. For patients requiring fewer passes (one or two), 12 had RBC-rich thrombi, and seven had fibrin-rich thrombi. For patients requiring three or more passes, 14 had RBC-rich thrombi, and 27 had fibrin-rich thrombi. The chi-square value for the association between the nature of the thrombus and the number of passes was 4.45, with a p-value of 0.035. Thus, a significant association was found between the nature of the thrombus and the number of passes.

## Discussion

Our study mainly points to a significant association between the histopathology of the thrombus retrieved from mechanical thrombectomy and the number of passes during the procedure. We noticed that thrombus components vary with the number of passes. The first two passes revealed higher erythrocyte and lower fibrin constituents compared to clots retrieved in three or more attempts. Clot pieces recovered following failed attempts were linked with more fibrin. These findings substantiate the role of fibrin and platelets in the gross adherence of the clot to retrieval devices. Our results validate the thrombus constituents' effect on the complexity of the thrombectomy procedure by analyzing clots retrieved from patients suffering from acute ischemic stroke.

Interestingly, we found a significant association between age and the nature of the thrombus. The age group 45-65 years tends to have more fibrin-containing clots compared to other age groups, leading to more difficult thrombus retrieval and requiring more passes. This finding may help identify a higher-risk population in mechanical thrombectomy. More research is needed to confirm these conclusions through multi-institutional studies with larger populations.

As our understanding of clot nature prior to interventional procedures is limited, uncertainties remain about whether a similar therapeutic technique is suitable for all thrombi. Substantial knowledge of the nature of clots is undoubtedly a key factor in the effectiveness of thrombectomy. Studies have histologically examined clot constituents and recorded pronounced variability [29-33]. Consistent with our observations, Duffy et al. outlined that clots retrieved in the first two passes are more likely to have higher erythrocyte content [34]. Later passes were primarily associated with fibrin-rich thrombi. These studies also observed that clots recovered after failed attempts had greater fibrin content.

Our study confirms that later passes are associated with more fibrin-rich clot fragments. Gunning et al. [35] disclosed that fibrin-dominant thrombi have a higher coefficient of friction than erythrocyte-dominant thrombi, which can increase the difficulty of retrieving fibrin-rich thrombi. Yoo et al. detailed that compression of fibrin-rich clots can result in a higher coefficient of friction, thus increasing the difficulty of thrombus retrieval and requiring multiple passes [33]. Gunning et al. described how fibrin-rich thrombi have a higher friction coefficient than RBC-rich thrombi, which complicates retrieval [35]. In a study of the fibrin-dominant clot, it was described that the subsequent compression of fibrin-dominant clots brought about a higher friction coefficient that might make the retrieval of the thrombus more complex when manifold passes are employed [33].

Various studies have outlined the relationship between clot nature and imaging traits. More dense artery signs on CT and vessel signs on MRI suggest a dominant erythrocyte clot, while the absence of these manifestations indicates fibrin-dominant thrombi. Inaccessible clots also have greater fibrin fractions than retrieved thrombi [36], appearing isodense on non-contrast CT scans [37]. Linking the imaging features of clots with their histopathology could help select more appropriate interventional techniques and predict reperfusion outcomes.

Some studies detail that achieving complete reperfusion from a single pass (First Pass Effect) is associated with significantly better patient outcomes. Additionally, O’Neill et al. reported that greater reperfusion speed and rates are achieved with fewer passes when aspiration is prioritized first [38]. Thus, identifying the nature of the thrombus beforehand may help clinicians achieve better patient outcomes with the first-pass effect. A previous study by Hao Y et al. observed an increased risk of symptomatic intracerebral hemorrhage in patients who underwent more than three retrieval attempts [39]. O. Mereuta et al. proposed that vessel wall injury is linked with soft thrombus texture, a greater number of recanalization maneuvers, and poor recanalization outcomes. However, this contradicts a previous study suggesting that harder thrombi are associated with more recanalization attempts and subsequent vessel wall injury. There may be a connection between the thrombus nature and vessel wall injury [40], which warrants further investigation in future studies. Interestingly, one study noted that more than three attempts might increase the reperfusion rate but not the likelihood of a favorable functional outcome [41]. Sporns et al. found that fibrin-dominant clots with low RBC percentages are significantly associated with longer procedure durations. Embolism during thrombectomy occurs more often with clots containing fewer erythrocytes and lower CT density, indicating greater fragility of these clots [22]. A thorough understanding of the nature of clots before the interventional procedure could improve patient selection and optimize therapeutic strategies. These studies highlight that thrombus composition may influence the number of passes and other factors.

This study has some limitations. First, it was a single-institution, retrospective study with a relatively small sample size. Other factors contributing to thrombus retrieval were not considered. A deeper understanding of clot nature might help identify potential reasons for unsuccessful reperfusion in some cases, aiding in the development of equipment that can retrieve tough thrombi more effectively with fewer passes. Future studies with larger sample sizes and considerations for confounding factors could further enhance our understanding of thrombus composition.

## Conclusions

This study demonstrates that RBC-dominant clots are associated with fewer passes. We also found that the age group 45-65 years tends to have fibrin-rich clots, which require more passes, making the procedure more challenging and leading to comparatively poorer patient outcomes. The insights gained from this study regarding the relationship between thrombus composition, the number of passes, and age may help in tailoring therapeutic techniques for different cases. Additionally, it highlights the need to improve existing technology to retrieve difficult clots in fewer passes, especially for patients for whom "time is brain."
